# Enzymatic Protein Immobilization on Amino-Functionalized Nanoparticles

**DOI:** 10.3390/molecules28010379

**Published:** 2023-01-02

**Authors:** Qun Ma, Boqiang He, Guojin Tang, Ran Xie, Peng Zheng

**Affiliations:** Key Laboratory of Coordination Chemistry, Chemistry and Biomedicine Innovation Center (ChemBIC), School of Chemistry and Chemical Engineering, Nanjing University, Nanjing 210023, China

**Keywords:** *Oa*AEP1, protein immobilization, enzymatic ligation, nanoparticle

## Abstract

The immobilization of proteins on nanoparticles has received much attention in recent years. Among different approaches, enzymatic protein immobilization shows unique advantages because of its site-specific connection. OaAEP1 is a recently engineered peptide ligase which can specifically recognize an *N*-terminal GL residue (*NH_2_*–Gly–Leu) and a C-terminal NGL amino acid residue (Asn–Gly–Leu–*COOH*) and ligates them efficiently. Herein, we report OaAEP1-mediated protein immobilization on synthetic magnetic nanoparticles. Our work showed that *Oa*AEP1 could mediate C-terminal site-specific protein immobilization on the amino-functionalized Fe_3_O_4_ nanoparticles. Our work demonstrates a new method for site-specific protein immobilization on nanoparticles.

## 1. Introduction

Nanoparticle-protein complexes have a wide range of applications, such as magnetic separation of proteins [1,2], enzyme-catalyzed proteolysis [3], drug delivery [4], and disease diagnosis [5]. Thus, efficient immobilization of proteins on nanoparticles has received increasing interest in recent years [6,7,8,9,10]. Many interactions, such as electrostatic interaction and covalent binding, were used [11,12,13]. However, these methods suffer from the disadvantages of non-specific binding and uncontrolled orientation [14]. Recently, the enzyme-mediated method could realize *N*- or C-terminal site-specific protein immobilization on the nanoparticle, and many related works were reported [14,15]. In 2020, the Francis group reported that proteins containing proline, thiol, or aniline functional groups were coupled to phenols-functionalized Au nanoparticles by enzyme tyrosinase using an oxidative coupling reaction [16]. The enzyme sortase, one of the transpeptidases, has often been used for versatile enzymatic protein immobilization with the requirements of the two substrates with a C-terminal LPxTG tag and an *N*-terminal glycine repeat [1,17,18,19,20,21,22,23].

*Oa*AEP1[C247A], cysteine 247 to alanine mutant of asparaginyl endopeptidedase 1 from *Oldenlandia affinis*, is a recently engineered protein ligase (abbreviated as *Oa*AEP1). It recognizes an *N*-terminal GL residue (*NH_2_*–Gly–Leu) and a C-terminal NGL amino acid residue (Asn–Gly–Leu–*COOH*) explicitly and ligates them efficiently. Thus, it is extensively used in protein/peptide ligation studies [24,25,26,27,28,29,30,31]. It is an excellent choice for protein immobilization, which has been well demonstrated for AFM-based single molecule-force spectroscopy (SMFS) studies of the immobilized protein [32,33,34,35].

**Scheme 1:**
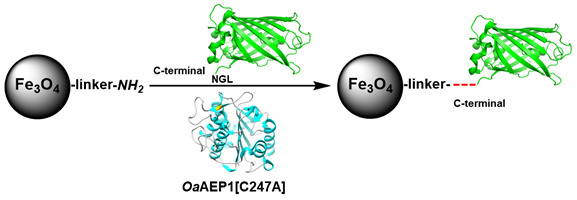
Schematic illustration of *Oa*AEP1 mediated protein immobilization on Fe_3_O_4_ nanoparticle.

In 2021, David J. Craik and Thomas Durek reported that the *Oa*AEP1-catalyzed peptide and protein were irreversibly labeled with various nonpeptidyl amine nucleophiles at a C-terminal asparagine of the peptide and protein. In their work, the protein eGFP with NGL at the C terminal could conjugate with the molecules containing primary amine nucleophiles, which was demonstrated by SDS-PAGE and ESI-MS [36]. Similar work reported on asparaginyl endopeptidase-mediated protein C-terminal ligation with amino-containing molecules [27]. Moreover, the Ploegh group reported *Oa*AEP1 catalyzed one-step protein modification on the surface of red blood cells [37]. Thus, we believe that this powerful enzymatic ligation can be adopted for protein immobilization on nanoparticles, and thus we used this method to immobilize an enhanced green fluorescent protein (eGFP) with C-terminal NGL residues on the amino-functionalized magnetic nanoparticle, abbreviated as Fe_3_O_4_-NH_2_ nanoparticle (Scheme 1).

## 2. Results and Discussions

### 2.1. Synthesis and Characterization of Fe_3_O_4_ Nanoparticles

**Figure 1:**
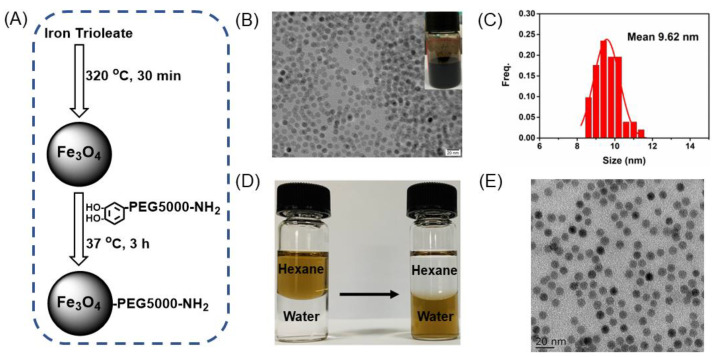
Characterizations of Fe_3_O_4_ nanoparticles. (**A**) Schematic description of the synthesis of oleic acid-capped hydrophobic Fe_3_O_4_–OA nanoparticles and catechol–PEG5000–NH_2_ modified hydrophilic Fe_3_O_4_–NH_2_ nanoparticles. (**B**) TEM image of Fe_3_O_4_–OA nanoparticles. Inset: photograph of the sample Fe_3_O_4_–OA nanoparticles dispersed in hexane. (**C**) The diameter distribution histogram of Fe_3_O_4_–OA nanoparticles. (**D**) Photograph of the Fe_3_O_4_ nanoparticles transferred from the organic phase to the aqueous phase. (**E**) TEM image of hydrophilic Fe_3_O_4_–NH_2_ nanoparticles.

First, the Fe_3_O_4_–OA nanoparticles were synthesized by the high-temperature pyrolysis of the precursor iron trioleate (Figure 1A) [38]. In the XRD spectra, the peak of the obtained sample (Figure S1) was found to be well-matched to the standard diffraction peaks of Fe_3_O_4_, indicating the formation of Fe_3_O_4_ [38,39,40]. The picture of Fe_3_O_4_–OA nanoparticles dispersed in cyclohexane (Figure 1B, inset) showed that the sample was homogeneous without precipitation. The TEM images (Figure 1B) showed that the Fe_3_O_4_–OA nanoparticles were monodisperse in cyclohexane with an average diameter of 9.62 nm (Figure 1C).

Then, catechol–PEG5000–NH_2_ was introduced and replaced oleic acid on the surface of Fe_3_O_4_ nanoparticles by the classic ligand exchange method to change Fe_3_O_4_ nanoparticles from the hydrophobic Fe_3_O_4_–OA to hydrophilic Fe_3_O_4_–NH_2_ based on the strong coordination of phenolic hydroxy groups of catechol with iron [41] (Figure 1A). As illustrated in Figure 1D, the Fe_3_O_4_ nanoparticles were transferred from the organic phase to the aqueous phase, indicating the transformation from the hydrophobic Fe_3_O_4_–OA to hydrophilic Fe_3_O_4_–NH_2_. The changes in the average diameter of Fe_3_O_4_–OA and Fe_3_O_4_–NH_2_ measured by dynamic light scattering (DLS) indicated that the modification of catechol–PEG5000–NH_2_ on the Fe_3_O_4_ nanoparticle was successful (Figure S2). Besides, the TEM image (Figure 1E) of the product Fe_3_O_4_–NH_2_ revealed that the Fe_3_O_4_–NH_2_ nanoparticles were similar to the Fe_3_O_4_–OA nanoparticles (Figure 1B). According to the amount of the nanoparticles and catechol–PEG5000–NH_2_, the amount of catechol–PEG5000–NH_2_ on the surface of 1 Fe_3_O_4_ nanoparticle was estimated as about 1200.

### 2.2. Immobilization of eGFP–ELP_8_–NGL on Fe_3_O_4_–NH_2_ Nanoparticle by OaAEP1

**Figure 2:**
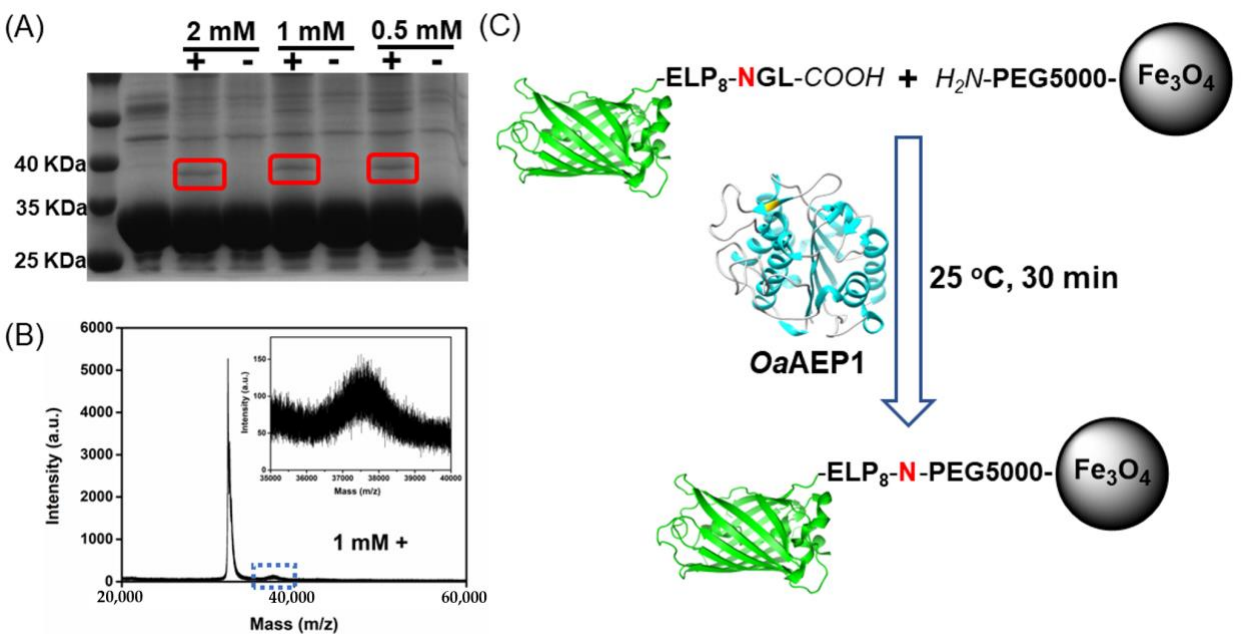
(**A**) SDS-PAGE gel result of the ligation of eGFP–ELP_8_–NGL with PEG5000–NH_2_ (2 mM; 1 mM; 0.5 mM) when *Oa*AEP1 was present and absent. (**B**) MALDI-TOF MS result of the ligation of eGFP–ELP_8_–NGL with PEG5000–NH_2_ (1 mM) in solution. (**C**) Schematic illustration of the immobilization of protein eGFP–ELP_8_–NGL on Fe_3_O_4_–NH_2_ nanoparticle by *Oa*AEP1.

First, the target protein eGFP–ELP_8_–NGL was constructed, showing an expected molecular weight of ~33 kDa from the SDS-PAGE gel experiment [41,42]. Then, we confirmed its ability to ligate with the corresponding protein GL–eGFP in a solution by *Oa*AEP1 using SDS-PAGE (Figure S3) [24,32]. Next, we examined the ability of eGFP–ELP_8_–NGL (50 μM) to conjugate with PEG5000–NH_2_ under various concentrations (2 mM; 1 mM; 0.5 mM). Notably, the eGFP–ELP_8_–NGL (50 μM), as the control group, is in the second lane. The SDS-PAGE gel result showed successful ligation, which is circled in red when *Oa*AEP1 is present (Figure 2A) [36]. Moreover, MALDI-TOF MS results revealed that eGFP–ELP_8_–NGL could conjugate with PEG5000–NH_2_ under a very low concentration of PEG5000–NH_2_ (0.05 mM) (Figure S4) [36]. We then used a 1 mM concentration of PEG5000–NH_2_ for all following experiments (Figure 2B).

**Figure 3:**
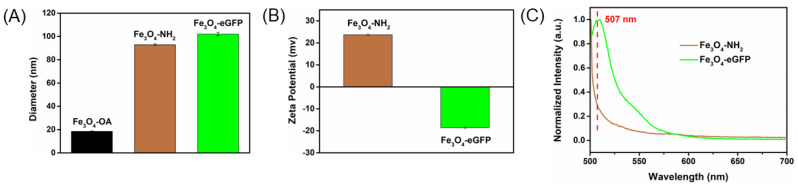
(**A**) Average diameter of Fe_3_O_4_–OA, Fe_3_O_4_–NH_2_, and Fe_3_O_4_–eGFP measured by DLS. (**B**) Zeta potential and (**C**) fluorescence emission spectra of Fe_3_O_4_–NH_2_ and Fe_3_O_4_–eGFP.

Finally, we immobilized eGFP–ELP_8_–NGL on a Fe_3_O_4_–NH_2_ nanoparticle by *Oa*AEP1 (Figure 2C). Furthermore, a number of characterizations were carried out to prove eGFP–ELP_8_–NGL’s conjugation on the Fe_3_O_4_–NH_2_ nanoparticle. First, the changes in average diameter of Fe_3_O_4_–NH_2_ and Fe_3_O_4_–eGFP measured by dynamic light scattering (DLS) along with zeta potential indicated that the conjugation of eGFP–ELP_8_–NGL with the Fe_3_O_4_–NH_2_ nanoparticle was successful (Figure 3A,B and Figure S5). Furthermore, the fluorescence emission spectra of Fe_3_O_4_–NH_2_ and Fe_3_O_4_–eGFP are depicted in Figure 3C. The fluorescence emission spectra of Fe_3_O_4_–eGFP ranged from 500 to 700 nm, with the emission peak located at 507 nm when excited at 488 nm, while the Fe_3_O_4_–NH_2_ exhibited almost negligible emission at a wavelength of 507 nm (Figure 3C and Figure S7).

To confirm the immobilization was mediated by *Oa*AEP1, not the non-specific interaction or electrostatic interaction, fluorescence emission spectroscopy measurement was performed. The group with *Oa*AEP1 showed an apparent fluorescence intensity compared with the group when *Oa*AEP1 was absent (Figure S6). It is worth noting that the two samples were washed with the dispersion solution three times and with washing buffer (50 mM Tris, 1 M NaCl, pH 7.0) five times, then dispersed in 0.5 mL dispersion buffer. In summary, all data above proved that the immobilization of protein eGFP–ELP_8_–NGL on Fe_3_O_4_–NH_2_ nanoparticles was successful. Moreover, considering the amount of protein eGFP–ELP_8_–NGL and the reaction efficiency, the number of proteins eGFP–ELP_8_–NGL on the Fe_3_O_4_–NH_2_ nanoparticle was estimated as about 20.

## 3. Materials and Methods

### 3.1. Materials

Iron trichloride hexahydrate, hexane, ethanol, and tetrahydrofuran (THF) were purchased from Sinopharm Chemical Reagent Co. Ltd. (Shanghai, China). Sodium oleate was purchased from Beijing InnoChem Science & Technology Co., Ltd. (Beijing, China). Oleic acid was purchased from Alfa Aesar (Shanghai, China). The 1-Octadecene was purchased from Acros Organics (Shanghai, China). Catechol–PEG5000–NH_2_ was purchased from Ponsure Biotechnology (Shanghai, China). Other reagents were purchased from Sangon Biotech Co. Ltd. (Shanghai, China). All reagents were used without further purification. Ultrapure water (18 MΩ cm^−1^) was obtained from a Millipore Milli-Q Advantage water purification system (Burlington, MA, USA). *E. coil BL21* (DE3) and XL1-Blue cells were purchased from TransGen Biotech Co. Ltd. (Beijing, China).

### 3.2. Sample Characterizations

X-ray diffraction (XRD) spectra were recorded on an X’TRA Powder diffractometer (Waltham, MA, USA). The size and morphology of nanoparticles were observed using a JEOL JEM-2100 transmission electron microscope (Tokyo, Japan) at 200 kV. Dynamic light scattering (DLS) was recorded at a wavelength of 659 nm, and zeta potential experiments were conducted on 90 Plus/BI-MAS equipment (Brookhaven, NY, USA). The concentration of Fe was determined by Atomic Absorption Spectroscopy on analytik jena novAA350 (Jena, Germany). The fluorescence spectra were measured on a HORIBA Jobin Yvon Fluoromax-4 fluorescence spectrometer (Irvine, CA, USA). Mass spectrometric analyses were performed using an UltrafleXtreme MALDI-TOF mass spectrometer (Bruker Daltonics, Billerica, MA, USA) operating in linear positive ion mode with Sinapinic acid (SA) as the matrix.

### 3.3. Protein Engineering

All the plasmids, except *Oa*AEP1, were based on the pET-28a vector, and all proteins were overexpressed in *E. coli* BL21(DE3) cells. *Oa*AEP1 was kindly provided by Dr. Wu, Bin. ELP is an elastin-like polypeptide [43,44]. The expression and purification details of *Oa*AEP1 can be found in our previous publications [45,46], and *Oa*AEP1 was exchanged into water (A_280_ = 1.46) by ultrafiltration. The genes of eGFP were purchased from Genscript (Nanjing, China). Construction of eGFP–ELP_8_–NGL and GL–eGFP was in the expression vector pET-28a by standard molecular biology and PCR techniques. The plasmids were transformed and then overexpressed in *E. coli* BL21 (DE3) cells. The bacteria kept in the LB medium containing 50 µg mL^−1^ kanamycin were grown to an OD600 = 0.6 and then were induced by 0.4 mM isopropyl β–d–thiogalactoside (IPTG) overnight at 16 °C. The cells were obtained by centrifugation at 9000 rpm for 5 min at 4 °C (Avanti JXN series, Beckman Coulter) (Brea, CA, USA). The cells were redispersed in dispersion buffer (50 mM Tris, 100 mM NaCl, pH 7.0) and lysed via a high-pressure homogenization. The supernatants were mixed with Co–NTA affinity beads and then kept for 3 h after the centrifugation operation at 30,000 rpm for 15 min. The mixture was washed with buffer (20 mM Tris, 400 mM NaCl, 2 mM imidazole, pH 7.0) several times and eluted in buffer (20 mM Tris, 400 mM NaCl, 250 mM imidazole, pH 7.0) immediately. The buffer of the protein solutions was all exchanged into dispersion buffer (50 mM Tris, 100 mM NaCl, pH 7.0) by ultrafiltration. The concentrations of eGFP–ELP_8_–NGL and GL–eGFP were 4.1 mg/mL and 4.8 mg/mL, respectively, which were determined by Nanodrop 2000 (Waltham, MA, USA).

### 3.4. Synthesis of Fe_3_O_4_–OA Nanoparticles

First, the precursor iron trioleate was synthesized. A 5.4 g amount of iron trichloride hexahydrate and 18.3 g of sodium oleate were dissolved in a mixed solvent composed of 40 mL ethanol, 30 mL distilled water, and 70 mL hexane. The resulting solution was heated to 70 °C and kept at 70 °C for 4 hours. When the reaction was completed, the upper organic layer containing the iron trioleate was washed with distilled water four times in a separatory funnel. Then, hexane was evaporated off, and iron trioleate in a waxy solid form was obtained. The iron trioleate was dissolved in 100 mL 1-Octadecene after drying in the oven for 24 h.

Fe_3_O_4_–OA nanoparticles were synthesized according to a previously published method with modification. Briefly, 1 mL oleic acid was added into 20 mL of iron trioleate solution above a three-necked flask, and then the mixture was heated to 320 °C and kept at 320 °C for 30 min. After cooling to room temperature, ethanol was added to the solution to precipitate the Fe_3_O_4_–OA nanoparticles. The Fe_3_O_4_–OA nanoparticles were washed with hexane and ethanol mixture several times and then dispersed in 10 mL hexane (~20 mg/mL).

### 3.5. Synthesis of Fe_3_O_4_–NH_2_ Nanoparticles

From the above hexane solution 1 mL was isolated then centrifuged and transferred into 0.4 mL tetrahydrofuran (THF). A 60 mg portion of catechol–PEG5000–NH_2_ was dissolved in 0.4 mL THF. The two solutions were mixed and placed in a shaker at 37 °C for 3 h. When the reaction was finished, hexane was added to precipitate the Fe_3_O_4_–NH_2_ nanoparticles. The acquired Fe_3_O_4_–NH_2_ nanoparticles were washed with THF and water three times and then dispersed in 5 mL dispersion solution (50 mM Tris, 100 mM NaCl, pH 7.0) for use.

### 3.6. Immobilization of eGFP–ELP_8_–NGL on Fe_3_O_4_–NH_2_ Nanoparticle by OaAEP1

Briefly, 0.5 mL eGFP–ELP_8_–NGL solution, 0.5 mL Fe_3_O_4_–NH_2_ dispersion, and 75 μL *Oa*AEP1 solution were mixed and placed in a shaker at 25 °C for 30 min. The reaction products were washed with the dispersion solution for three times and buffer (50 mM Tris, 1 M NaCl, pH 7.0) five times and then dispersed in 0.5 mL dispersion solution ([Fe] 31 mM).

## 4. Conclusions

In summary, we developed a new enzymatic method for protein immobilization on nanoparticles using *Oa*AEP1. The target protein eGFP with a C-terminal NGL peptide tag was immobilized on the Fe_3_O_4_–NH_2_ nanoparticle. Considering the easy access of PEG–NH_2_ molecules or molecules with a primary amine, we believe this method can be useful for immobilizing other important proteins on nanoparticles and applied for further applications.

## Data Availability

All data are presented in the manuscript.

## Supplementary Materials

The following supporting information can be downloaded at: https://www.mdpi.com/article/10.3390/molecules28010379/s1. Figure S1: XRD patterns of the obtained Fe_3_O_4_ and standard diffraction peaks of Fe_3_O_4_ (JCPDS NO.19-0629); Figure S2: Average diameter of Fe_3_O_4_-OA and Fe_3_O_4_-NH_2_ measured by DLS; Figure S3: SDS-PAGE gel result of the ligation of eGFP-ELP_8_-NGL with protein GL-eGFP by *Oa*AEP1; Figure S4: MALDI-TOF MS result of the ligation of eGFP-ELP_8_-NGL with PEG5000-NH_2_ (A, E 1 mM; B, F 0.5 mM; C, G 0.1 mM; D, H 0.05 mM) when *Oa*AEP1 was present (upper) and absent (bottom); Figure S5: Hydrodynamic diameter distribution of Fe_3_O_4_-OA, Fe_3_O_4_-NH_2_, Fe_3_O_4_-eGFP; Figure S6: The fluorescence emission spectra of Fe_3_O_4_-NH_2_ (black) and the obtained Fe_3_O_4_-eGFP complex when *Oa*AEP1 was present (blue) and absent (red); Figure S7: The fluorescence emission spectrum of target protein eGFP-ELP_8_-NGL excited at 488 nm, which is same as free eGFP.

## Sample Availability

Samples of all protein-coated nanoparticles are available from the authors.
