# Supplementary material for: Enzymatic Protein Immobilization on Amino-Functionalized Nanoparticles
Source: Molecules. 2023 Jan 2;28(1):379. doi: 10.3390/molecules28010379 (PMC9822503; doi:10.3390/molecules28010379)
Supplement: Supplementary file 1 [file molecules-28-00379-s001.zip › Supporting information.pdf]

Supporting information

Enzymatic Protein Immobilization on Amino-functionalized Nanoparticles

Estimation of the number of proteins immobilized on nanoparticles:

The total mass of Fe_3O_4: m= 12 mg.

Assuming the Fe_3O_4 nanoparticle is a regular sphere, the mass of a single Fe_3O_4 nanoparticle: $m_{single} = \rho * V = 5.18\ g/cm^3 \times (4/3) \times \pi \times (9.62\ nm/2)^3 = 2.4*10^{-15}$ mg.

The total Fe_3O_4 nanoparticles is: $N_{total} = 12\ mg / 2.4*10^{-15}\ mg = 5*10^{15}$.

The molar amount of catechol-PEG5000-NH_2: n = 55 mg/5500 g/moL = 0.01 mmoL.

The concentration of catechol-PEG5000-NH_2: c = 0.01 mmoL /$5*10^{-3}$ L = 2 mM.

The number of catechol-PEG5000-NH_2: $N_{PEG} = 0.01\ mmoL*N_A*10^{-3} = 6.02*10^{18}$.

The number of catechol-PEG5000-NH_2 on a Fe_3O_4 nanoparticle: $N_{particle} = 6.02*10^{18} / 5*10^{15} = 1.2*10^3$.

The number of eGFP-ELP_8-NGL on a Fe_3O_4 nanoparticle: $N_{protein}= 1.2*10^3*2*10^{-2} = 24$.

Protein sequence

eGFP-ELP_8-NGL (MW: ~33 kDa)

MGHHHHHHMVSKGEELFTGVVPILVELDGDVNGHKFSVSGEGEGDATYGKL
TLKFICTTGKLPVPWPTLVTTLTYGVQCFSRYPDHMKQHDFFKSAMPEGYVQ
ERTIFFKDDGNYKTRAEVKFEGDTLVNRIELKGIDFKEDGNILGHKLEYNYNS
HNVYIMADKQKNGIKVNFKIRHNIEDGSVQLADHYQQNTPIGDGPVLLPDNH
YLSTQSALSKDPNEKRDHMVLLEFVTAAGITLGMDELYKRSVPGVGVPGVGV
PGEGVPGVGVPGVGVPGVGVPGVGVPGEGVPGGLRSNGL

The fluorescence emission spectrum excited at 488 nm was shown in figure S7.

GL-eGFP (MW: ~29 kDa)

MGLHHHHHHGSMVSKGEELFTGVVPILVELDGDVNGHKFSVSGEGEGDATY
GKLTLKFICTTGKLPVPWPTLVTTLTYGVQCFSRYPDHMKQHDFFKSAMPEG
YVQERTIFFKDDGNYKTRAEVKFEGDTLVNRIELKGIDFKEDGNILGHKLEYN
YNSHNVYIMADKQKNGIKVNFKIRHNIEDGSVQLADHYQQNTPIGDGPVLLP
DNHYLSTQSALSKDPNEKRDHMVLLEFVTAAGITLGMDELYKRS

Supplementary Figures

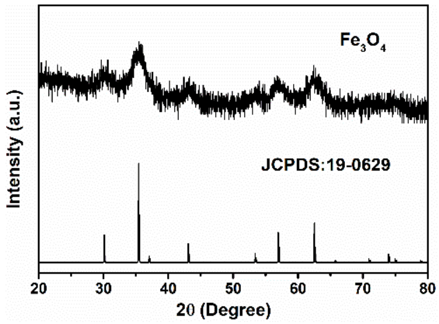

Figure S1. XRD patterns of the obtained Fe_3O_4 and standard diffraction peaks of Fe_3O_4 (JCPDS NO.19-0629).

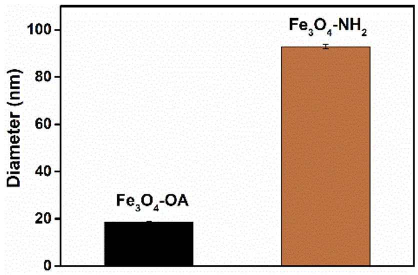

Figure S2. Average diameter of Fe_3O_4-OA and Fe_3O_4-NH_2 measured by DLS.

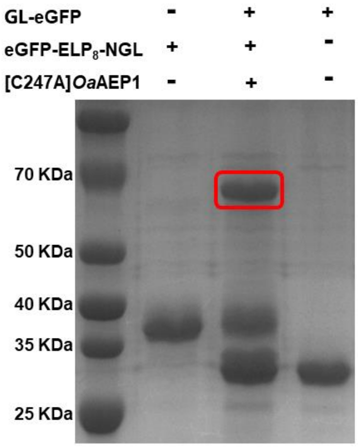

Figure S3. SDS-PAGE gel result of the ligation of eGFP-ELP$_8$-NGL with protein GL-eGFP by *Oa*AEP1.

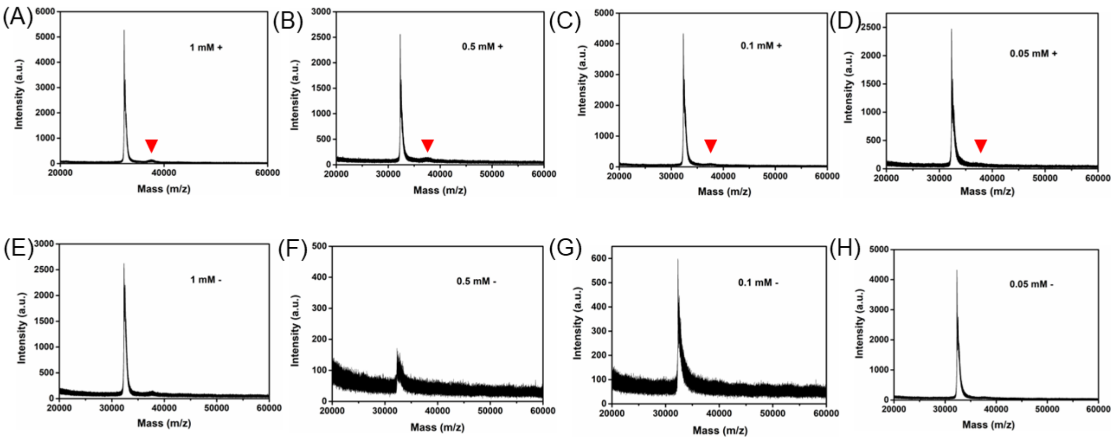

Figure S4. MALDI-TOF MS result of the ligation of eGFP-ELP8-NGL with PEG5000-NH_2 (A, E 1 mM; B, F 0.5 mM; C, G 0.1 mM; D, H 0.05 mM) when *Oa*AEP1 was present (upper) and absent (bottom).

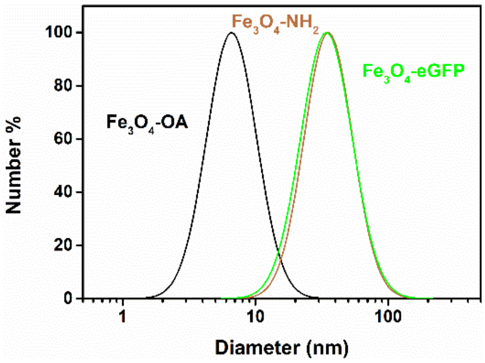

Figure S5. Hydrodynamic diameter distribution of Fe_3O_4-OA, Fe_3O_4-NH_2, Fe_3O_4-eGFP.

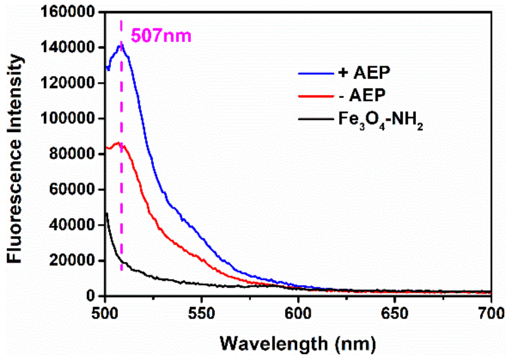

Figure S6. The fluorescence emission spectra of Fe_3O_4-NH_2 (black) and the obtained Fe_3O_4-eGFP complex when *Oa*AEP1 was present (blue) and absent (red).

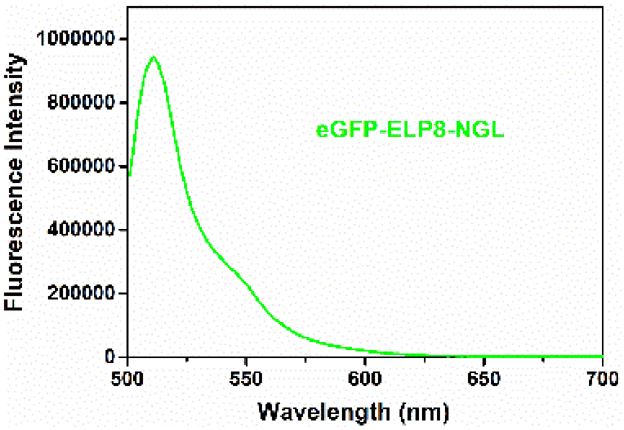

Figure S7. The fluorescence emission spectrum of target protein eGFP-ELP$_8$-NGL excited at 488 nm, which is same as free eGFP.
